# Supplementary material for: Comparative Effectiveness Study of Home-Based Interventions to Prevent CA-MRSA Infection Recurrence
Source: Antibiotics (Basel). 2021 Sep 13;10(9):1105. doi: 10.3390/antibiotics10091105 (PMC8465828; doi:10.3390/antibiotics10091105)
Supplement: Supplementary file 1 [file antibiotics-10-01105-s001.zip › antibiotics-1349704-supplementary.pdf]

Supplementary Materials

Table S1. Abbreviations Used.

Acronym	Definition
AIDS	Acquired Immunodeficiency Syndrome
CA-MRSA	Community-Associated Methicillin-Resistant Staphylococcus Aureus
CA-MSSA	Community-Associated Methicillin-Sensitive Staphylococcus Aureus
CAMP1	Community-Acquired MRSA Project
CBPR	Community-based Participatory Research
CDN	Clinical Directors Network
CHW	Community Health Workers
CLSI	Clinical and Laboratory Standards Institute
CON	Control Group
COPD	Chronic Obstructive Pulmonary Disease
CVA	Cerebrovascular Accident
ED	Emergency Department
EHR	Electronic Health Record
ER	Emergency Room
EXP	Experimental Group
FQHC	Federally Qualified Health Center
HA-MRSA	Healthcare-Associated MRSA
HTE	Heterogeneity of Treatment Effects
HIV	Human Immunodeficiency Virus
I&D	Incision and Drainage
ICU	Intensive Care Unit
ITT	Intent to Treat
MNAR	Missing Not at Random
MRSA	Methicillin-Resistant *Staphylococcus aureus*
PBRN	Practice-based Research Network
QoL	Quality of Life
RCT	Randomized Clinical Trial
SSTI	Skin and Soft Tissue Infection
TIA	Transient Ischemic Attack
UC	Usual Care

Table S2. Characteristics of consented participants who completed the baseline home visit (T1) versus patients who did not complete the baseline home visit (T1).

Variable	Completed T1 (n = 119)			Did Not Complete T1 (n = 66)			*p*-Value
	TOTAL (n = 119)	EXP (n = 63)	UC (n = 56)	TOTAL (n = 66)	EXP (n = 33)	UC (n = 33)	
**Age**							
**Mean age (SD)**	38.1 (14.9)	39.5 (15.4)	36.5 (14.4)	40.7 (17.4)	42.4 (17.3)	39.0 (17.6)	0.29
**Median**	37.0	38.0	36.0	40.5	45.0	35.2	
**IQR**	27, 51	27, 54	27, 48	25.4, 54.8	27.6, 58.8	24.3, 52.5	
**Gender, n (%)**							
**Female**	47 (39.5)	22 (34.9)	25 (44.6)	20 (30.3)	13 (39.4)	7 (21.2)	0.21
**Male**	72 (60.5)	41 (65.1)	31 (55.4)	46 (69.7)	20 (60.6)	26 (78.8)	
**Ethnicity: Hispanic/Latino, n (%)**	72 (64.9)	37 (62.7)	35 (67.3)	18 (69.2)	10 (71.4)	9 (64.3)	0.91
**Race, n (%)**							
**Black/African American**	27 (22.7)	14 (22.2)	13 (23.2)	14 (21.2)	10 (30.3)	4 (12.1)	
**White**	22 (18.5)	13 (20.6)	9 (16.1)	6 (9.1)	3 (9.1)	3 (9.1)	
**American Indian/Alaska Native**	1 (0.8)	0 (0)	1 (1.8)	1 (1.5)	0 (0)	1 (3.0)	<0.0001
**Asian**	0 (0)	0 (0)	0 (0)	2 (3.0)	2 (6.1)	0 (0)	
**More than one race/Other**	21 (17.6)	11 (17.5)	10 (17.9)	17 (25.8)	6 (18.2)	11 (33.3)	
**Did not Report**	48 (40.3)	25 (39.7)	23 (41.1)	26 (39.4)	12 (36.4)	14 (42.4)	
**Baseline MRSA/MSSA**							
**MRSA+**	59 (50.0)	41 (66.1)	18 (32.1)	35 (53.0)	19 (57.6)	16 (48.5)	0.65
**MSSA+**	59 (50.0)	21 (33.9)	38 (67.9)	33 (50.0)	15 (45.4)	18 (55.6)	0.96
**Incision & Drainage (I&D)**	70 (61.9)	34 (56.7)	36 (67.9)	37 (60.7)	18 (58.1)	19 (63.3)	0.87

**Recruitment Site: ED vs. FQHC**							
**ED**	102 (85.7)	54 (85.7)	85.71 (48)	57 (86.4)	54 (85.7)	48 (85.7)	0.90
**FQHC**	17 (14.3)	9 (14.3)	14.29 (8)	9 (13.6)	9 (14.3)	8 (14.3)	
**Lesion Location**							
**Abdomen/Torso**	7.56 (9)	6.35 (4)	8.93 (5)	16.67 (11)	15.15 (5)	18.18 (6)	0.72
**Arm**	6.72 (8)	7.94 (5)	5.36 (3)	4.55 (3)	3.03 (1)	6.06 (2)	
**Axilla**	16.81 (20)	15.87 (10)	17.86 (10)	9.09 (6)	15.15 (5)	3.03 (1)	
**Back**	3.36 (4)	3.17 (2)	3.53 (2)	4.55 (3)	6.06 (2)	3.03 (1)	
**Breast**	0.84 (1)	1.59 (1)	0	0	0	0	
**Buttock**	13.45 (16)	11.11 (7)	16.07 (9)	13.64 (9)	6.06 (2)	21.21 (7)	
**Foot/Ankle**	12.61 (15)	9.52 (6)	16.07 (9)	13.64 (9)	15.15 (5)	12.12 (4)	
**Groin**	4.20 (5)	3.17 (2)	5.36 (3)	1.52 (1)	0	3.03 (1)	
**Hand/Finger**	8.40 (10)	7.94 (4)	8.93 (5)	7.58 (5)	12.12 (4)	3.03 (1)	
**Head/Neck**	8.40 (10)	9.52 (6)	7.14 (4)	10.61 (7)	9.09 (3)	12.12 (4)	
**Lower Leg**	9.24 (11)	14.29 (9)	3.57 (2)	12.12 (8)	9.09 (3)	15.15 (5)	
**Thigh**	8.40 (10)	9.52 (6)	7.14 (4)	6.06 (4)	9.09 (3)	3.03 (1)	
**Lesion Size**							
**0–5 cm**	90.76 (108)	87.30 (55)	94.64 (53)	80.30 (53)	76.79 (26)	81.82 (27)	0.13
**>5 to 10 cm**	6.72 (8)	9.52 (6)	3.57 (2)	18.18 (12)	21.21 (7)	15.15 (5)	
**>10 to 15 cm**	1.68 (2)	3.17 (2)	0	1.52 (1)	0	3.03 (1)	
**over 15 cm**	0.84 (1)	0	1.79 (1)	0	0	0	
**Signs and Symptoms of SSTI**							
**Redness**	109 (91.6)	58 (92.1)	51 (91.1)	60 (90.9)	29 (87.9)	31 (93.9)	0.87
**Swelling**	107 (89.9)	56 (88.9)	51 (91.1)	59 (89.4)	33 (84.9)	31 (93.9)	0.91

**Warmth**	91 (76.5)	49 (77.8)	42 (75.0)	49 (74.2)	20 (60.6)	29 (87.9)	0.74
**Pain/Tenderness**	110 (92.4)	59 (93.7)	51 (91.1)	61 (92.4)	30 (90.9)	31 (93.9)	0.998
**Complaint of "Spider Bite"**	5 (4.2)	4 (6.4)	1 (1.8)	0 (0)	0 (0)	0 (0)	0.09
**Redness**	109 (91.6)	58 (92.1)	51 (91.1)	60 (90.9)	29 (87.9)	31 (93.9)	0.87
**Lesion Type**							
**Folliculitis**	10 (8.4)	6 (9.5)	4 (7.1)	8 (12.1)	3 (9.1)	5 (15.2)	
**Abscess**	92 (77.3)	46 (73.0)	46 (82.1)	50 (75.8)	24 (72.7)	26 (78.8)	
**Furuncle/Boil**	5 (4.2)	2 (3.2)	3 (5.4)	3 (4.6)	1 (3.0)	2 (6.1)	0.68
**Carbuncle**	4 (3.4)	1 (1.6)	3 (5.4)	2 (3.0)	1 (3.0)	1 (3.0)	
**Cellulitis**	23.53 (28)	18 (28.6)	10 (17.9)	20 (30.3)	9 (27.3)	33.33 (11)	
**Purulence**							
**Fluctuance**	72 (60.5)	33 (52.9)	39 (69.6)	40 (60.6)	17 (51.5)	23 (69.7)	
**Yellow/White Center**	44 (37.0)	21 (33.3)	23 (41.1)	37 (56.1)	16 (48.5)	21 (63.6)	
**Central Point or Head**	45 (37.8)	21 (33.3)	24 (42.9)	32 (48.5)	15 (45.5)	17 (51.5)	0.43
**Draining Pus**	62 (52.1)	32 (50.8)	30 (53.6)	35 (53.0)	16 (48.5)	19 (57.6)	
**Possible to aspirate with needle and syringe**	24 (20.2)	9 (14.3)	15 (26.8)	11 (16.7)	5 (15.2)	6 (18.2)	
**Follow-up Visit**							
**Yes**	99 (88.4)	51 (87.9)	48 (88.9)	54 (88.5)	27 (87.1)	27 (90.0)	0.98
**Treatment**							
**Incision and Drainage**	73 (61.9)	35 (56.5)	38 (67.9)	37 (60.7)	18 (58.1)	19 (63.3)	
**Antibiotic prescription, any type**	102 (86.4)	54 (87.1)	48 (85.7)	56 (84.9)	28 (84.9)	28 (84.9)	0.94
**Both**	61 (51.7)	29 (46.8)	32 (57.1)	33 (48.5)	16 (48.5)	16 (48.5)	

**Nasal Surveillance**							
**MRSA+**	16 (19.5)	8 (19.5)	8 (19.5)	12 (23.1)	7 (26.9)	5 (19.2)	0.41
**MSSA+**	33 (40.2)	17 (41.5)	16 (39.0)	15 (28.9)	9 (34.6)	6 (23.1)	

Table S3. Demographics of participants who were followed-up vs. not followed-up.

	FOLLOWED-UP (N = 92)	NOT FOLLOWED-UP (N = 28)	*P*-VALUE
**MEAN AGE (SD)**	38.9 (15.5)	35.7 (12.6)	0.31
**GENDER, N (%)**			
**FEMALE**	36 (39.6)	11 (37.9)	1.00
**MALE**	55 (60.4)	18 (62.1)	
**ETHNICITY: HISPANIC/LATINO, N (%)**	62 (72.1)	10 (37.0)	0.0006
**RACE, N (%)**			
**BLACK/AFRICAN AMERICAN**	17 (32.7)	10 (52.6)	0.32
**WHITE**	16 (30.8)	6 (31.6)	
**AMERICAN INDIAN/ALASKA NATIVE**	1 (1.9)	0 (0)	
**ASIAN**	0 (0)	0 (0)	
**MORE THAN ONE RACE/OTHER**	18 (34.6)	3 (15.8)	
**BASELINE MRSA/MSSA**			
**MRSA+**	47 (51.7)	12 (41.4)	0.40
**MSSA+**	44 (48.4)	17 (58.6)	0.40
**INCISION & DRAINAGE (I&D)**	55 (64.0)	15 (53.6)	0.37
**RECRUITED FROM ED VS. FQHC**			
**ED**	76 (83.5)	27 (93.1)	0.24
**FQHC**	15 (16.5)	2 (6.9)	

Table S4. Microbiologic and dermatologic characteristics of study participants at baseline.

Dermatologic Characteristics	Total (n = 119)	Experimental (n = 63)	Usual Care (n = 56)
Wound, n (%)			
MRSA+	59 (50.0)	41 (66.1)	18 (32.1)
MSSA+	59 (50.0)	21 (33.9)	38 (67.9)
USA 300	50 (42.4)	32 (51.6)	18 (32.1)
Non-USA 300	51 (43.2)	24 (38.7)	27 (48.2)
Nasal, n (%)			
MRSA+	16 (19.5)	8 (19.5)	8 (19.5)
MSSA+	33 (40.2)	17 (41.5)	16 (39.0)
USA 300	36 (30.5)	23 (37.1)	13 (23.2)
Non-USA 300	46 (39.0)	24 (38.7)	22 (39.3)
Location of Lesion, n (%)			
Abdomen/Torso	9 (7.6)	4 (6.5)	5 (8.9)
Arm	8 (6.8)	5 (8.1)	3 (5.4)
Axilla	20 (17.0)	10 (16.1)	10 (17.9)
Back	4 (3.4)	2 (3.2)	2 (3.6)
Breast	1 (0.9)	1 (1.6)	0 (0)
Buttock	16 (13.6)	7 (11.3)	9 (16.1)
Foot/Ankle	15 (12.7)	6 (9.7)	9 (16.1)
Groin	5 (4.2)	2 (3.2)	3 (5.4)
Hand/Finger	9 (7.6)	4 (6.5)	5 (8.9)

Head/Neck	10 (8.5)	6 (9.7)	4 (7.1)
Lower Leg	11 (9.3)	9 (14.5)	2 (3.6)
Thigh	10 (8.5)	6 (9.7)	4 (7.1)
Size, n (%)			
0–5 cm	95 (89.6)	49 (86.0)	46 (93.9)
>5 to 10 cm	8 (7.6)	6 (10.5)	2 (4.1)
>10 to 15 cm	2 (1.9)	2 (3.5)	0 (0)
over 15 cm	1 (0.9)	0 (0)	1 (2.0)
Mean (SD)	4.05 ± 2.55	3.14 ± 2.83	3.64 ± 2.71
Median (IQR)	4 (3, 5)	3 (2, 4)	3 (2, 4)
Signs/Symptoms, n (%)			
Redness	108 (91.5)	57 (91.9)	51 (91.1)
Swelling	106 (89.8)	55 (88.7)	51 (91.1)
Warmth	90 (76.3)	48 (77.4)	42 (75.0)
Pain/Tenderness	109 (92.4)	58 (93.6)	51 (91.1)
Complaint of "Spider Bite"	5 (4.2)	4 (6.5)	1 (1.8)
Lesion Type, n (%)			
Folliculitis	10 (8.5)	6 (9.7)	4 (7.1)
Abscess	91 (77.1)	45 (72.6)	46 (82.1)
Furuncle/Boil	5 (4.2)	2 (3.2)	3 (5.4)
Carbuncle	4 (3.4)	1 (1.6)	3 (5.4)
Cellulitis	28 (23.7)	18 (29.0)	10 (17.9)
Purulence, n (%)			
Fluctuance	71 (60.2)	32 (51.6)	39 (69.6)
Yellow/White Center	43 (36.4)	20 (32.3)	23 (41.1)
Central Point or Head	45 (38.1)	21 (33.9)	24 (42.9)
Draining Pus	62 (52.5)	32 (51.6)	30 (53.6)
Possible to aspirate with needle and syringe	24 (20.3)	9 (14.5)	15 (26.8)
Follow-Up Visit, n (%)			
Yes	98 (88.3)	50 (87.7)	48 (88.9)
Treatment, n (%)			
Incision and Drainage	73 (61.9)	35 (56.5)	38 (67.9)
Antibiotic prescription	102 (86.4)	54 (87.1)	48 (85.7)
Both	61 (51.7)	29 (46.8)	32 (57.1)
Wound Antibiogram (Resistance), n (%)			
Ceftaroline	0 (0)	0 (0)	0 (0)
Ciprofloxacin	35 (30.4)	23 (37.7)	12 (22.2)
Clindamycin	13 (11.3)	7 (11.5)	6 (11.1)
Erythromycin	65 (56.5)	40 (65.6)	25 (46.3)
Gentamicin	6 (5.2)	4 (6.6)	2 (3.7)
Levofloxacin	29 (25.7)	18 (30.5)	11 (30.4)
Oxacillin	56 (49.1)	40 (66.7)	16 (29.6)
Penicillin	115 (100.0)	60 (100.0)	54 (100.0)
Rifampin	1 (0.9)	0 (0)	1 (1.9)
Tetracycline	7 (6.1)	2 (3.3)	5 (9.26)
Tigecycline	0 (0)	0 (0)	0 (0)
Trimethoprim/Sulfamethoxazole	2 (1.7)	2 (3.3)	0 (0)
Vancomycin	0 (0)	0 (0)	0 (0)

Table S5. Patient reported outcome scores at baseline and six months for the total sample, experimental condition, and control condition.

	Total Sample (n = 119) Mean ± SD		Experimental (n = 63) Mean ± SD		Usual Care (n = 56) Mean ± SD		*p*-Value [a]
	Baseline (n = 119)	Six-months (n = 92)	Baseline (n = 63)	Six-months (n = 49)	Baseline (n = 56)	Six-months (n = 43)	
Pain Interference	15.74 ± 7.95	9.17 ± 5.01	17.30 ± 8.09	9.42 ± 4.62	13.95 ± 7.49	8.87 ± 5.49	0.25
Emotional Distress–Depression Short Form	13.40 ± 6.93	11.16 ± 5.51	13.74 ± 7.12	11.48 ± 5.24	13.00 ± 6.76	10.77 ± 5.88	0.96
Satisfaction with Care	26.62 ± 18.03	25.13 ± 15.48	26.35 ± 19.87	25.00 ± 17.66	26.89 ± 19.06	25.27 ± 15.72	0.93
Satisfaction with Participation in Social Roles	25.49 ± 8.50	30.15 ± 6.25	24.70 ± 9.06	29.65 ± 6.63	26.38 ± 7.82	30.75 ± 5.80	0.80
Prevention Self-Efficacy	33.89 ± 5.01	34.54 ± 5.30	34.17 ± 5.62	34.73 ± 5.72	33.56 ± 4.27	34.31 ± 4.81	0.87
Decision-making autonomy	84.22 ± 21.31	91.33 ± 14.65	85.28 ± 21.06	92.90 ± 13.91	83.04 ± 21.78	89.35 ± 15.51	0.72
Infection Prevention and Hygiene Behaviors	5.1 ± 1.17	4.64 ± 0.90	5.06 ± 1.14	4.63 ± 1.06	5.21 ± 1.21	4.64 ± 0.67	0.32

Note: [a] *p*-value refers to dependent *t*-tests examining changes over time (baseline-six-month follow up) by condition (experimental versus control).

Table S6. Measures, data sources and time points [a].

	Construct/Topic	Measure/Instrument	Data Source	Time Point
**Primary Outcome**				
**A**	Recurrence	Chart Review, Clinical Response Questionnaire [1]	EHR, Survey	T4
**Secondary/Intermediate Outcomes**				
**B**	Pain Interference	PROMIS Adult Pain Interference [2]	Survey	T0, T4
**C**	Depression	PROMIS Adult Depression Short Form [3]	Survey	T0, T4
**D**	Quality of Life	Quality of Life Scale, Medical Outcomes Trust [4]	Survey	T0
**E**	Satisfaction with Care	AHRQ CAHPS 2.0 12-month 4pt Scale [5]	Survey	T0, T4
**F**	Household Contamination	Microbiologic Data (culture, sensitivity), Environmental Surface Contamination and Household Member Colonization [6]	Laboratory, Survey	T1, T3
**G**	Household Transmission	Index Patient Household Member SSTI Report, Household Member SSTI History [7]	Survey	T1, T3, T4
**Co-variates, Effect Moderators**				
**H**	Bacterial Genotype and Phenotype	Dermatological Symptoms Instrument [1] Molecular Data (*Spa*, MLST, *mecA*, SCC*mec*, ACME, *pvl*, *mupA*) [6]	Laboratory, Clinician Report	T0, T1, T3
**I**	Infection Prevention and Hygiene Behaviors	Personal Hygiene Score [7]	Survey	T1, T2, T3, T4

**J**	Household Crowding	Household Crowding Index [7,8]	Survey	T1, T3
**K**	Household Sharing	Household Sharing Index [9]	Survey	T0, T1, T3
**L**	Social Network and Environmental Exposures	Social and Environmental Exposures Questionnaire	Survey	T0
**M**	Prevention Self-Efficacy	Generalized Self-Efficacy Scale [9,10]	Survey	T0, T4
**N**	Decision-making autonomy	O'Connor AM. User Manua—Decision Self-Efficacy [11]	Survey	T0, T4
**O**	Satisfaction with Participation in Social Roles	PROMIS Satisfaction with Social Roles and Activities [12]	Survey	T0, T4
**P**	Overall Medication Adherence	Morisky 8-Item Medication Adherence Questionnaire, Adapted [13]	Survey	T0, T2, T4
**Q**	Modified Morisky-Mupirocin Adherence	Morisky 8-Item Medication Adherence Questionnaire, Adapted [13]	Survey	T0, T2
**R**	Modified Morisky-Chlorhexidine Adherence	Morisky 8-Item Medication Adherence Questionnaire, Adapted [13]	Survey	T0, T2

Note: T0 and T1 = baseline; T2 = one month post T1; T3 = three months post T1; T4 = 6 months post T1. [a] Information reflects a combination of details from cited sources as well adapted versions. A certified translation service provided forward- and back-translation on measures that were not available in Spanish.

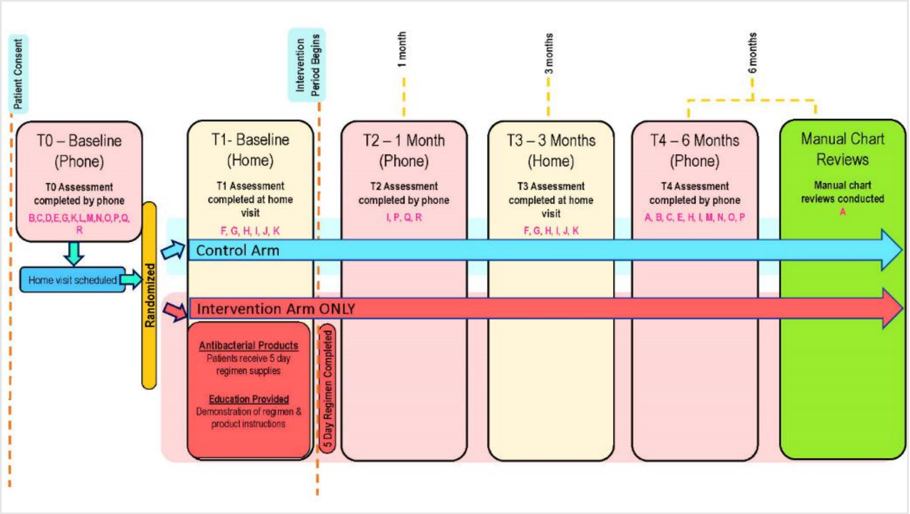

Figure S1. Study procedures and timeline. * Letters refer to measures described in Table S6.

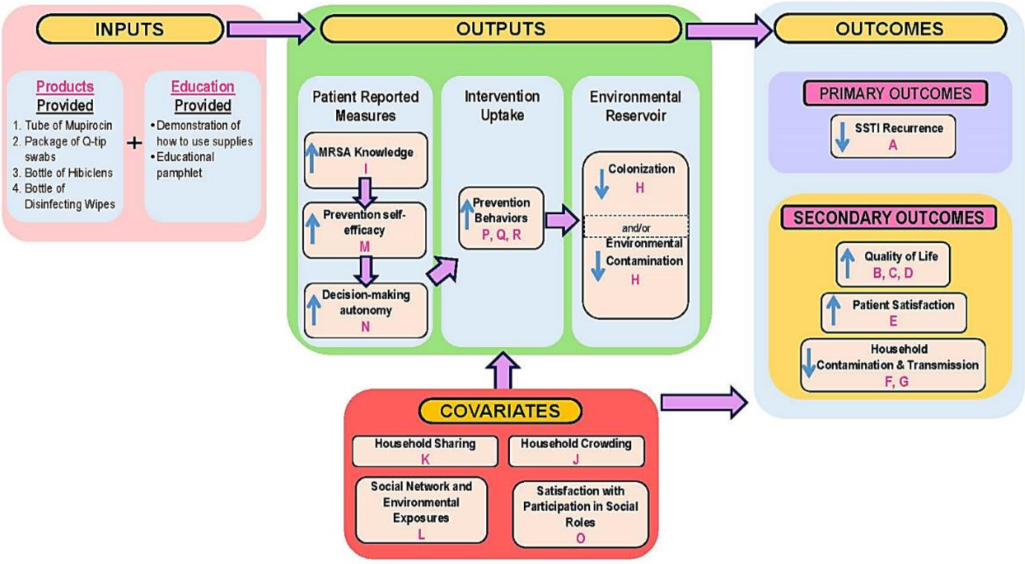

Figure S2. Conceptual model. * Letters refer to measures described in Table S6.

Reference

1. Forcade, N.A.; Parchman, M.L.; Jorgensen, J.H.; Du, L.C.; Nyren, N.R.; Trevino, L.B.; Pena, J.; Mann, M.W.; Munoz, A.; Trevino, S.B.; et al. Prevalence, severity, and treatment of community-acquired methicillin-resistant *Staphylococcus aureus* (CA-MRSA) skin and soft tissue infections in 10 medical clinics in Texas: A South Texas Ambulatory Research Network (STARNet) study. *J. Am. Board Fam. Med.* **2011**, *24*, 543–550.
2. Amtmann, D.A.; Cook, K.F.; Jensen, M.P.; Chen, W.-H.; Choi, S.W.; Revicki, D.; Cella, D.; Rothrock, N.; Keefe, F.; Callahan, L.; et al. Development of a PROMIS item bank to measure pain interference. *Pain* **2010**, *146*, 173–182.
3. Pilkonis, P.A.; Yu, L.; Dodds, N.E.; Johnston, K.L.; Maihoefer, C.C.; Lawrence, S.M. Validation of the depression item bank from the Patient-Reported Outcomes Measurement Information System (PROMIS) in a three-month observational study. *J. Psychiatr. Res.* **2014**, *56*, 112–119.
4. Ware, J.E., Jr.; Sherbourne, C.D. The MOS 36-item short-form health survey (SF-36). I. Conceptual framework and item selection. *Med. Care* **1992**, *30*, 473–483.
5. Agency for Healthcare Research and Quality. CAHPS Clinician & Group 12-Month Survey 2.0. Published 2017. Accessed February 2, 2019. http://www.ahrq.gov/cahps/surveys- guidance/cg/12-month/index.html
6. Pardos de la Gandara, M.; Raygoza Garay, J.A.; Mwangi, M.; Tobin, J.N.; Tsang, A.; Khalida, C.; D'Orazio, B.; Kost, R.G.; Leinberger-Jabari, A.; Coffran, C.; et al. Molecular types of methicillin-resistant *Staphylococcus aureus* and methicillin-sensitive *Staphylococcus aureus* strains causing skin and soft tissue infections and

nasal colonization, identified in community health centers in New York City. *J. Clin. Microbiol.* **2015**, *53*, 2648–2658.
7. Miller, L.G.; Quan, C.; Shay, A.; Mostafaie, K.; Bharadwa, K.; Tan, N.; Matayoshi, K.; Cronin, J.; Tan, J.; Tagudar, G.; et al. A prospective investigation of outcomes after hospital discharge for endemic, community-acquired methicillin-resistant and -susceptible *Staphylococcus aureus* skin infection. *Clin. Infect. Dis.* **2007**, *44*, 483–492.
8. Melki, I.S.; Beydoun, H.A.; Khogali, M.; Tamim, H.; Yunis, K.A. Household crowding index: A correlate of socioeconomic status and inter-pregnancy spacing in an urban setting. *J. Epidemiol. Community Health* **2004**, *58*, 476–480.
9. Jones, D.L.; Ishii Owens, M.; Lydston, D.; Tobin, J.N.; Brondolo, E.; Weiss, S.M. Self-efficacy and distress in women with AIDS: The SMART/EST women's project. *AIDS Care* **2010**, *22*, 1499–1508.
10. Schwarzer, R.; Jerusalem, M. Generalized Self-Efficacy Scale. In *Measures in Health Psychology: A User's Portfolio*; NFER-Nelson: Windsor, UK, 1995; pp. 35–37.
11. O'Connor, A.M. *User Manual—Decision Self-Efficacy Scale*; Ottawa Hospital Research Institute: Ottawa, ON, Canada, 1995.
12. Hahn, E.A.; DeWalt, D.A.; Bode, R.K.; Garcia, S.F.; DeVellis, R.F.; Correia, H.; Cella, D. New English and Spanish social health measures will facilitate evaluating health determinants. *Health Psychol.* **2014**, *33*, 490–499.
13. Morisky, D.E.; Green, L.W.; Levine, D.M. Concurrent and predictive validity of a self-reported measure of medication adherence. *Med. Care* **1986**, *24*, 67–74.
